# (2-Amino-7-methyl-4-oxidopteridine-6-carboxyl­ato-κ^3^ *O* ^4^,*N* ^5^,*O* ^6^)aqua­(ethane-1,2-diamine-κ^2^ *N*,*N*′)nickel(II) dihydrate

**DOI:** 10.1107/S160053681300069X

**Published:** 2013-01-12

**Authors:** Siddhartha S. Baisya, Parag S. Roy

**Affiliations:** a Department of Chemistry, University of North Bengal, Siliguri 734 013, India

## Abstract

The Ni^II^ atom in the title complex, [Ni(C_8_H_5_N_5_O_3_)(C_2_H_8_N_2_)(H_2_O)]·2H_2_O, is six-coordinated in a distorted octa­hedral geometry by a tridentate 2-amino-7-methyl-4-oxidopteridine-6-carboxyl­ate (pterin) ligand, a bidentate ancillary ethane-1,2-diamine (en) ligand and a water mol­ecule. The pterin ligand forms two chelate rings. The en and pterin ligands are arranged nearly orthogonally [dihedral angle between the mean plane of the en mol­ecule and the pterin ring = 77.1 (1)°]. N—H⋯O, O—H⋯N and O—H⋯O hydrogen bonds link the complex mol­ecules and lattice water mol­ecules into a three-dimensional network. π–π inter­actions are observed between the pyrazine and pyrimidine rings [centroid–centroid distance = 3.437 (2) Å].

## Related literature

For the importance of pterin in metalloenzymes, see: Basu & Burgmayer (2011 ▶); Burgmayer (1998 ▶); Fitzpatrick (2003 ▶); Fukuzumi & Kojima (2008 ▶); Kaim *et al.* (1999 ▶). For the structure of a related nickel complex, see: Crispini *et al.* (2005 ▶). For structures of related copper complexes, see: Odani *et al.* (1992 ▶). For the electron-shuffling ability of the pterin unit as well as its donor groups and the effect on the geometric parameters of related complexes, see: Beddoes *et al.* (1993 ▶); Kohzuma *et al.* (1988 ▶); Russell *et al.* (1992 ▶). For the synthesis of the pterin ligand, see: Wittle *et al.* (1947 ▶). For refinement of H atoms, see: Cooper *et al.* (2010 ▶).
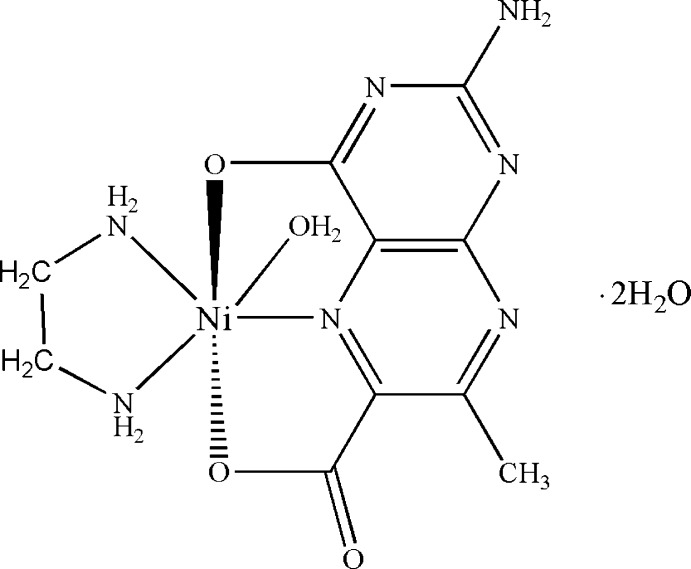


## Experimental

### 

#### Crystal data

[Ni(C_8_H_5_N_5_O_3_)(C_2_H_8_N_2_)(H_2_O)]·2H_2_O
*M* *_r_* = 392.01
Monoclinic, 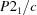

*a* = 10.406 (4) Å
*b* = 14.323 (5) Å
*c* = 10.450 (4) Å
β = 93.294 (6)°
*V* = 1554.9 (10) Å^3^
*Z* = 4
Mo *K*α radiation
μ = 1.29 mm^−1^
*T* = 293 K
0.49 × 0.38 × 0.28 mm

#### Data collection

Bruker Kappa APEXII CCD diffractometer
Absorption correction: multi-scan (*SADABS*; Sheldrick, 1996 ▶) *T*_min_ = 0.56, *T*_max_ = 0.70
8393 measured reflections
3488 independent reflections
2760 reflections with *I* > 2σ(*I*)
*R*_int_ = 0.035

#### Refinement

*R*[*F* ^2^ > 2σ(*F* ^2^)] = 0.050
*wR*(*F* ^2^) = 0.135
*S* = 0.92
3488 reflections
217 parameters
H-atom parameters constrained
Δρ_max_ = 1.12 e Å^−3^
Δρ_min_ = −0.84 e Å^−3^

### 

Data collection: *APEX2* (Bruker, 2007 ▶); cell refinement: *SAINT* (Bruker, 2007 ▶); data reduction: *SAINT*; program(s) used to solve structure: *SHELXS97* (Sheldrick, 2008 ▶); program(s) used to refine structure: *CRYSTALS* (Betteridge *et al.*, 2003 ▶); molecular graphics: *CAMERON* (Watkin *et al.*, 1996 ▶); software used to prepare material for publication: *CRYSTALS*.

## Supplementary Material

Crystal structure: contains datablock(s) global, I. DOI: 10.1107/S160053681300069X/hy2612sup1.cif

Structure factors: contains datablock(s) I. DOI: 10.1107/S160053681300069X/hy2612Isup2.hkl

Additional supplementary materials:  crystallographic information; 3D view; checkCIF report

## Figures and Tables

**Table 1:** Hydrogen-bond geometry (Å, °)

*D*—H⋯*A*	*D*—H	H⋯*A*	*D*⋯*A*	*D*—H⋯*A*
N1—H192⋯O1^i^	0.89	2.39	3.175 (4)	147
N1—H192⋯O2^i^	0.89	2.42	3.243 (4)	154
N2—H221⋯O4	0.87	2.40	3.103 (5)	137
N2—H222⋯O6^ii^	0.85	2.22	3.064 (4)	176
N7—H171⋯O2^iii^	0.92	2.16	2.890 (4)	136
N7—H172⋯O5^iv^	0.96	2.29	3.225 (5)	167
O4—H231⋯O3^ii^	0.83	1.89	2.683 (4)	160
O4—H232⋯O5^v^	0.82	2.04	2.858 (5)	171
O5—H242⋯O1	0.83	2.21	3.010 (4)	160
O6—H181⋯O4	0.82	1.96	2.774 (4)	171
O6—H182⋯N5^iv^	0.84	2.06	2.805 (3)	148

Symmetry codes: (i) 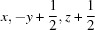; (ii) 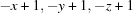; (iii) 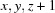; (iv) 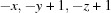; (v) 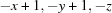.

**Fig. 1.**
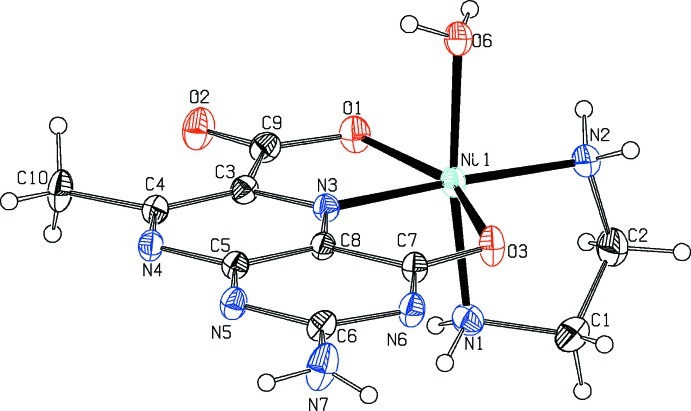
The molecular structure of the title compound. Displacement ellipsoids are drawn at the 40% probability level. Lattice water molecules are omitted for clarity.

**Fig. 2.**
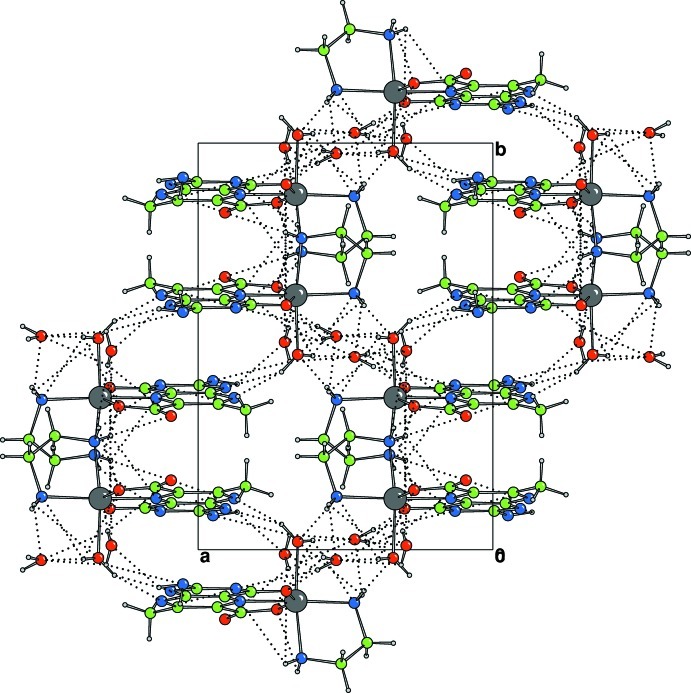
The crystal packing diagram of the title compound, viewed along the c axis. Dotted lines indicate hydrogen bonds.

## supplementary crystallographic information

### Comment

The importance of pterins in several classes of metalloenzymes has catalysed symbiotic developments of their coordination chemistry (Basu & Burgmayer, 2011; Burgmayer, 1998; Fitzpatrick, 2003; Fukuzumi & Kojima, 2008; Kaim *et al.*, 1999). A SciFinder search reveals the existence of only one structurally characterized nickel(II)–pterin complex (Crispini *et al.*, 2005), thereby highlighting the urgency of development in this direction. The present endeavour is concerned with the title complex, possessing both a tridentate pterin ligand and a σ-donor ligand like en. The six-coordinated Ni^II^ atom shows departure from a regular octahedral geometry with respect to both bond lengths and angles (Fig. 1). The equatorial plane is formed by the two N atoms (N1, N2) of en, the pyrazine ring N atom (N3) of the pterin ligand and the aqua O atom (O6). The axial positions are occupied by the two pterin O atoms (O1 and O3), with the latter one forming the longest axial bond [2.327 (2) Å]. One important factor causing distortion from regular octahedral geometry is that this pterin ligand forms two five-membered chelate rings with small bite angles [76.31 (9) and 77.20 (10)°], instead of only one per pterin ligand for the earlier case (Crispini *et al.*, 2005). A perusal of the charge balance of this complex indicates that this pterin ligand acts as a binegative tridentate ONO-donor. A near orthogonal disposition of the en ligand and pterin chelate ring is observed, which helps to minimize the steric repulsion. Of the three axes, least deviation from linearity is observed in the N3—Ni1—N2 direction [177.56 (11)°], where the highest electron density is concentrated [Ni1—N3 = 1.976 (2), Ni1—N2 = 2.065 (3) Å]. It represents the unique combination of a σ-donor atom N2 (en) and the N3 atom of the redox noninnocent pterin ligand from the opposite directions of the Ni^II^ centre (d^8^), with possible assistance from the π-donating phenolate and carboxylate O atoms (Kohzuma *et al.*, 1988). Again, location of the pyrazine ring N atom (N3) in the equatorial plane is consistent with the earlier observations on related copper complexes (Odani *et al.*, 1992).

Although the exocyclic bond length data of the pyrazine ring, *e.g.* C3—C9 [1.527 (4) Å] and C4—C10 [1.503 (4) Å] reflect only limited conjugation with the pyrazine ring π system, the corresponding bond length data of the pyrimidine ring, C7—O3 [1.267 (3) Å] and C6—N7 [1.349 (4) Å] merit attention. Small deviations, *e.g.* 2.02° and 1.37° of the C7/N6/C6 and C5/N5/C6 segments respectively, with respect to the C6—N7 multiple bond, indicate near planarity for the pyrimidine ring. So it can participate in the electron-shuffling process by the pterin unit from the pyrazine ring N4 to the C7-carbonyl group, as per literature suggestion (Beddoes *et al.*, 1993; Russell *et al.*, 1992). Formation of the Ni1—O3 bond assists this process.

In the crystal, the complex molecules and lattice water molecules are linked by intermolecular N—H···O, O—H···N and O—H···O hydrogen bonds (Table 1) into a three-dimensional network. The lattice water molecules are decisive for the crystal packing (Figure 2).

### Experimental

2-Amino-4-hydroxy-7-methylpteridine-6-carboxylic acid sesquihydrate (C_8_H_7_N_5_O_3_.1.5H_2_O) was obtained by published procedure (Wittle *et al.*, 1947). The title complex was prepared by the slow addition of an aqueous alkaline solution (NaOH: 44 mg, 1.1 mmol) of the pterin ligand (124 mg, 0.5 mmol) to a well stirred warm (323 K; paraffin oil bath) aqueous reaction mixture containing NiSO_4_.7H_2_O (140 mg, 0.5 mmol) and 1,2-ethanediamine (36 mg, 0.6 mmol) under subdued light; final volume was 35 ml. The pH value was adjusted to 9.2 and the stirring was continued for 3 h. Upon standing, the reaction medium deposited yellow-brown crystals after 2 days, which were suitable for single-crystal X-ray diffraction (yield: 30%). Analytically pure compound could be obtained by filtration, repeated washing with small quantities of water and drying *in vacuo* over silica gel. Analysis, calculated for C_10_H_19_N_7_NiO_6_: C 30.70, H 4.89, N 25.06%; found: C 30.51, H 5.11, N 24.55%.

### Refinement

H atoms were all located in a difference map, but those attached to C atoms were repositioned geometrically. The H atoms were initially refined with soft restraints on bond lengths and angles to regularize their geometry (C—H = 0.93–0.98, N—H = 0.86–0.89, O—H = 0.82 Å) and with *U*_iso_(H) = 1.2–1.5*U*_eq_(parent atom), after which the positions were refined with riding constraints (Cooper *et al.*, 2010).

### Crystal data

**Table:**

[Ni(C_8_H_5_N_5_O_3_)(C_2_H_8_N_2_)(H_2_O)]·2H_2_O	*F*(000) = 816
*M**_r_* = 392.01	*D*_x_ = 1.675 Mg m^−^^3^
Monoclinic, *P*2_1_/*c*	Mo *K*α radiation, λ = 0.71073 Å
Hall symbol: -P 2ybc	Cell parameters from 8393 reflections
*a* = 10.406 (4) Å	θ = 2.0–28.2°
*b* = 14.323 (5) Å	µ = 1.29 mm^−^^1^
*c* = 10.450 (4) Å	*T* = 293 K
β = 93.294 (6)°	Plate, brown
*V* = 1554.9 (10) Å^3^	0.49 × 0.38 × 0.28 mm
*Z* = 4	

### Data collection

**Table:**

Bruker Kappa APEXII CCD diffractometer	2760 reflections with *I* > 2σ(*I*)
Graphite monochromator	*R*_int_ = 0.035
φ & ω scans	θ_max_ = 28.2°, θ_min_ = 2.0°
Absorption correction: multi-scan (*SADABS*; Sheldrick, 1996)	*h* = −13→13
*T*_min_ = 0.56, *T*_max_ = 0.70	*k* = −18→15
8393 measured reflections	*l* = −11→13
3488 independent reflections	

### Refinement

**Table:**

Refinement on *F*^2^	Primary atom site location: structure-invariant direct methods
Least-squares matrix: full	Hydrogen site location: difference Fourier map
*R*[*F*^2^ > 2σ(*F*^2^)] = 0.050	H-atom parameters constrained
*wR*(*F*^2^) = 0.135	Method = Modified Sheldrick *w* = 1/[σ^2^(*F*^2^) + (0.09*P*)^2^ + 1.95*P*], where *P* = (max(*F*_o_^2^,0) + 2*F*_c_^2^)/3
*S* = 0.92	(Δ/σ)_max_ = 0.001
3488 reflections	Δρ_max_ = 1.12 e Å^−^^3^
217 parameters	Δρ_min_ = −0.84 e Å^−^^3^
0 restraints	

### Special details

**Table:**

Experimental. The crystal was placed in the cold stream of an Oxford Cryosystems open-flow nitrogen cryostat (Cosier, J. & Glazer, A.*M*., 1986. J. Appl. Cryst. 105–107) with a nominal stability of 0.1 K.

### Fractional atomic coordinates and isotropic or equivalent isotropic displacement parameters (Å^2^)

**Table:**

	*x*	*y*	*z*	*U*_iso_*/*U*_eq_	
Ni1	0.33168 (3)	0.37414 (3)	0.40998 (3)	0.0270	
O1	0.2677 (2)	0.35342 (17)	0.2158 (2)	0.0355	
C9	0.1450 (3)	0.3461 (2)	0.1944 (3)	0.0297	
O2	0.0914 (2)	0.3290 (2)	0.0880 (2)	0.0430	
C3	0.0659 (3)	0.3591 (2)	0.3117 (3)	0.0255	
N3	0.1423 (2)	0.37361 (17)	0.4173 (2)	0.0245	
C8	0.0909 (3)	0.3850 (2)	0.5295 (3)	0.0236	
C5	−0.0427 (3)	0.3859 (2)	0.5402 (3)	0.0248	
N4	−0.1228 (2)	0.37363 (19)	0.4340 (2)	0.0282	
C4	−0.0692 (3)	0.3588 (2)	0.3213 (3)	0.0279	
C10	−0.1616 (3)	0.3442 (3)	0.2074 (3)	0.0400	
H111	−0.1653	0.2810	0.1853	0.0637*	
H113	−0.1353	0.3768	0.1326	0.0631*	
H112	−0.2477	0.3644	0.2257	0.0629*	
N5	−0.0930 (2)	0.39909 (19)	0.6560 (2)	0.0285	
C6	−0.0042 (3)	0.4045 (2)	0.7565 (3)	0.0290	
N6	0.1283 (2)	0.4046 (2)	0.7564 (2)	0.0310	
C7	0.1792 (3)	0.3970 (2)	0.6422 (3)	0.0268	
O3	0.2995 (2)	0.39847 (18)	0.6257 (2)	0.0369	
N7	−0.0522 (3)	0.4136 (3)	0.8732 (3)	0.0489	
O6	0.3383 (2)	0.51989 (16)	0.3730 (2)	0.0341	
H182	0.2833	0.5626	0.3735	0.0551*	
H181	0.3889	0.5230	0.3156	0.0554*	
N1	0.3513 (3)	0.2334 (2)	0.4537 (3)	0.0343	
C1	0.4854 (4)	0.2201 (3)	0.5050 (4)	0.0463	
C2	0.5735 (3)	0.2713 (3)	0.4201 (4)	0.0475	
N2	0.5298 (3)	0.36927 (19)	0.4057 (3)	0.0343	
H221	0.5559	0.3888	0.3326	0.0516*	
H222	0.5630	0.3994	0.4691	0.0520*	
H211	0.6626	0.2705	0.4560	0.0603*	
H212	0.5706	0.2394	0.3383	0.0606*	
H202	0.5096	0.1546	0.5123	0.0585*	
H201	0.4910	0.2482	0.5908	0.0590*	
H192	0.2966	0.2195	0.5135	0.0560*	
H191	0.3371	0.2006	0.3817	0.0561*	
O4	0.5313 (3)	0.5259 (3)	0.2007 (3)	0.0699	
H231	0.5947	0.5499	0.2395	0.1050*	
H232	0.5749	0.5189	0.1384	0.1048*	
O5	0.2936 (3)	0.4894 (2)	−0.0008 (3)	0.0646	
H241	0.3113	0.5370	0.0413	0.1023*	
H242	0.3059	0.4542	0.0622	0.1021*	
H171	0.0041	0.4206	0.9433	0.0500*	
H172	−0.1306	0.4416	0.8977	0.0500*	

### Atomic displacement parameters (Å^2^)

**Table:**

	*U*^11^	*U*^22^	*U*^33^	*U*^12^	*U*^13^	*U*^23^
Ni1	0.0198 (2)	0.0344 (2)	0.0270 (2)	−0.00059 (15)	0.00148 (14)	−0.00027 (15)
O1	0.0291 (12)	0.0516 (15)	0.0262 (11)	−0.0017 (10)	0.0057 (9)	−0.0032 (10)
C9	0.0317 (16)	0.0329 (16)	0.0248 (14)	0.0026 (12)	0.0035 (12)	0.0003 (12)
O2	0.0388 (13)	0.0651 (17)	0.0246 (11)	0.0035 (12)	−0.0024 (9)	−0.0070 (11)
C3	0.0270 (15)	0.0261 (15)	0.0230 (13)	0.0008 (11)	−0.0010 (11)	−0.0025 (11)
N3	0.0232 (12)	0.0270 (12)	0.0234 (12)	−0.0004 (9)	0.0012 (9)	−0.0012 (9)
C8	0.0213 (13)	0.0270 (15)	0.0225 (13)	0.0001 (11)	0.0002 (10)	0.0002 (11)
C5	0.0243 (14)	0.0264 (15)	0.0236 (13)	0.0007 (11)	0.0010 (11)	0.0007 (11)
N4	0.0210 (12)	0.0360 (14)	0.0273 (12)	−0.0003 (10)	−0.0011 (9)	0.0011 (10)
C4	0.0254 (14)	0.0316 (16)	0.0261 (14)	−0.0005 (11)	−0.0026 (11)	0.0014 (12)
C10	0.0275 (16)	0.062 (2)	0.0293 (16)	0.0013 (15)	−0.0087 (13)	−0.0057 (15)
N5	0.0222 (12)	0.0391 (15)	0.0242 (12)	0.0033 (10)	0.0026 (9)	−0.0006 (10)
C6	0.0281 (15)	0.0344 (16)	0.0245 (14)	0.0061 (12)	0.0006 (11)	0.0019 (12)
N6	0.0258 (13)	0.0416 (15)	0.0252 (12)	0.0018 (11)	−0.0016 (10)	−0.0017 (11)
C7	0.0233 (14)	0.0311 (16)	0.0255 (14)	0.0000 (11)	−0.0018 (11)	−0.0014 (12)
O3	0.0213 (11)	0.0557 (15)	0.0332 (12)	−0.0005 (10)	−0.0013 (9)	−0.0076 (10)
N7	0.0346 (16)	0.090 (3)	0.0223 (13)	0.0139 (16)	0.0031 (11)	−0.0031 (15)
O6	0.0233 (10)	0.0379 (13)	0.0413 (12)	0.0031 (9)	0.0028 (9)	0.0008 (10)
N1	0.0297 (14)	0.0376 (15)	0.0361 (14)	−0.0018 (11)	0.0058 (11)	0.0034 (12)
C1	0.042 (2)	0.047 (2)	0.049 (2)	0.0061 (16)	−0.0049 (16)	0.0111 (17)
C2	0.0299 (18)	0.047 (2)	0.065 (2)	0.0032 (15)	−0.0009 (17)	0.0042 (19)
N2	0.0263 (13)	0.0369 (15)	0.0396 (15)	−0.0014 (11)	0.0009 (11)	0.0022 (12)
O4	0.0535 (18)	0.117 (3)	0.0386 (15)	−0.0370 (18)	−0.0034 (13)	0.0002 (16)
O5	0.065 (2)	0.0592 (19)	0.071 (2)	0.0075 (15)	0.0150 (16)	0.0168 (16)

### Geometric parameters (Å, º)

**Table:**

Ni1—O1	2.120 (2)	C6—N7	1.350 (4)
Ni1—N3	1.977 (3)	N6—C7	1.338 (4)
Ni1—O3	2.324 (2)	C7—O3	1.273 (4)
Ni1—O6	2.125 (2)	N7—H171	0.917
Ni1—N1	2.075 (3)	N7—H172	0.958
Ni1—N2	2.066 (3)	O6—H182	0.838
O1—C9	1.288 (4)	O6—H181	0.821
C9—O2	1.239 (4)	N1—C1	1.478 (5)
C9—C3	1.527 (4)	N1—H192	0.892
C3—N3	1.339 (4)	N1—H191	0.892
C3—C4	1.416 (4)	C1—C2	1.503 (5)
N3—C8	1.326 (4)	C1—H202	0.974
C8—C5	1.401 (4)	C1—H201	0.981
C8—C7	1.461 (4)	C2—N2	1.479 (5)
C5—N4	1.361 (4)	C2—H211	0.980
C5—N5	1.359 (4)	C2—H212	0.969
N4—C4	1.349 (4)	N2—H221	0.872
C4—C10	1.501 (4)	N2—H222	0.847
C10—H111	0.934	O4—H231	0.828
C10—H113	0.965	O4—H232	0.821
C10—H112	0.970	O5—H241	0.827
N5—C6	1.361 (4)	O5—H242	0.833
C6—N6	1.378 (4)		
			
O1—Ni1—N3	77.16 (10)	C5—N5—C6	114.6 (2)
O1—Ni1—O3	153.46 (8)	N5—C6—N6	129.4 (3)
N3—Ni1—O3	76.30 (9)	N5—C6—N7	115.6 (3)
O1—Ni1—O6	88.57 (10)	N6—C6—N7	115.0 (3)
N3—Ni1—O6	93.08 (9)	C6—N6—C7	116.6 (2)
O3—Ni1—O6	92.13 (9)	C8—C7—N6	117.7 (3)
O1—Ni1—N1	95.53 (11)	C8—C7—O3	118.1 (3)
N3—Ni1—N1	94.21 (11)	N6—C7—O3	124.2 (3)
O3—Ni1—N1	87.12 (10)	Ni1—O3—C7	109.05 (19)
O6—Ni1—N1	172.28 (9)	C6—N7—H171	118.7
O1—Ni1—N2	103.50 (11)	C6—N7—H172	130.3
N3—Ni1—N2	177.63 (11)	H171—N7—H172	104.8
O3—Ni1—N2	103.04 (10)	Ni1—O6—H182	133.3
O6—Ni1—N2	89.22 (10)	Ni1—O6—H181	102.4
N1—Ni1—N2	83.47 (11)	H182—O6—H181	115.6
Ni1—O1—C9	115.57 (18)	Ni1—N1—C1	106.5 (2)
O1—C9—O2	124.2 (3)	Ni1—N1—H192	108.2
O1—C9—C3	115.1 (3)	C1—N1—H192	110.2
O2—C9—C3	120.6 (3)	Ni1—N1—H191	108.4
C9—C3—N3	111.1 (3)	C1—N1—H191	110.2
C9—C3—C4	129.8 (3)	H192—N1—H191	113.2
N3—C3—C4	119.1 (3)	N1—C1—C2	108.6 (3)
C3—N3—Ni1	120.9 (2)	N1—C1—H202	112.7
C3—N3—C8	119.9 (3)	C2—C1—H202	110.7
Ni1—N3—C8	119.15 (19)	N1—C1—H201	106.5
N3—C8—C5	121.6 (3)	C2—C1—H201	109.5
N3—C8—C7	117.4 (3)	H202—C1—H201	108.8
C5—C8—C7	121.0 (3)	C1—C2—N2	109.2 (3)
C8—C5—N4	119.9 (3)	C1—C2—H211	111.3
C8—C5—N5	120.5 (3)	N2—C2—H211	109.2
N4—C5—N5	119.6 (3)	C1—C2—H212	107.6
C5—N4—C4	117.9 (3)	N2—C2—H212	111.4
C3—C4—N4	121.6 (3)	H211—C2—H212	108.2
C3—C4—C10	122.6 (3)	C2—N2—Ni1	109.3 (2)
N4—C4—C10	115.9 (3)	C2—N2—H221	106.5
C4—C10—H111	110.4	Ni1—N2—H221	111.7
C4—C10—H113	112.1	C2—N2—H222	107.1
H111—C10—H113	106.2	Ni1—N2—H222	109.2
C4—C10—H112	111.0	H221—N2—H222	112.9
H111—C10—H112	108.2	H231—O4—H232	88.8
H113—C10—H112	108.8	H241—O5—H242	93.4

### Hydrogen-bond geometry (Å, º)

**Table:**

*D*—H···*A*	*D*—H	H···*A*	*D*···*A*	*D*—H···*A*
N1—H192···O1^i^	0.89	2.39	3.175 (4)	147
N1—H192···O2^i^	0.89	2.42	3.243 (4)	154
N2—H221···O4	0.87	2.40	3.103 (5)	137
N2—H222···O6^ii^	0.85	2.22	3.064 (4)	176
N7—H171···O2^iii^	0.92	2.16	2.890 (4)	136
N7—H172···O5^iv^	0.96	2.29	3.225 (5)	167
O4—H231···O3^ii^	0.83	1.89	2.683 (4)	160
O4—H232···O5^v^	0.82	2.04	2.858 (5)	171
O5—H242···O1	0.83	2.21	3.010 (4)	160
O6—H181···O4	0.82	1.96	2.774 (4)	171
O6—H182···N5^iv^	0.84	2.06	2.805 (3)	148

Symmetry codes: (i) *x*, −*y*+1/2, *z*+1/2; (ii) −*x*+1, −*y*+1, −*z*+1; (iii) *x*, *y*, *z*+1; (iv) −*x*, −*y*+1, −*z*+1; (v) −*x*+1, −*y*+1, −*z*.
